# Iowa State Medical Society

**Published:** 1869-04

**Authors:** Philip Harvey, A. G. Field

**Affiliations:** President, Burlington; Secretary, Des Moines


### IOWA STATE MEDICAL SOCIETY.

The Seventeenth Annual Meeting of the Iowa State Medical Society will be held in the city of Des Moines, commencing at ten o'clock A. M., on Wednesday, the 26th day of May, 1869. We herewith append a full list of the committees as forwarded to us by the Secretary, and trust that every member whose name appears will heartily co-operate with the chairman of his committee in furnishing material for able reports upon the various subjects before the Society.

The following Committees are expected to report:

*On Surgery*—Drs. J. C. Hughes, C. H. Rawson, G. R. Henry.

*On Epidemics*—Drs. A. M. Carpenter, J. C. Stone, M. B. Cochran.

*On Criminal Abortion*—Drs. J. H. Robinson, Wm. Watson, Wm. Voght.

*On Diseases of Children*—Drs. S. B. Thrall, D. V. Cole, S. B. Cherry.

*On Medical Jurisprudence*—Drs. T. J. Saunders, F. Loyd, N. L. Smith.

*On Puerperal Diseases*—Drs. H. T. Cleaver, T. J. Caldwell, D. Hutchinson.

*On New Remedies*—Drs W. W. Nassau, William H. Gibbon, G. M. Staples.

*On Ophthalomology*—Drs. A. W. McCluer, J. C. Hughes, E. H. Hazen.

*On New Instruments and Devices for Diagnosis*—Drs. J. C. Blackburn, A. G. Field, L. French.

*On Physical Exploration*—Drs. J. C. Schrader, Wm. McClelland, Wm. Gutch.

*On Diseases of the Urinary Organs*—Drs. W. W. Rauseau, D. E. Beadle, J. W. Gustine.

*On Medical Inhalations*—Drs. J. W. H. Baker, J. W. Grayham, D. C. Roundy.

*On the Uses and Abuses of Mercury*—Drs. F. Loyd, J. S. Maxwell. J. M. Wetherwax.

*On Special Surgery*—Drs. W. F. Peck, W. H. Gibbon, H. Heed.

*On Hypodermic Injections*—Drs. J. C. Stone, W. W. Nassau, D. W. Stewart.

*On Insanity*—Drs. J. Williamson, S. H. Sawyers, N. Udell.

*On Anesthetics*—Drs. N. Bird, M. Cousins, T. H. Elder.

*On Orthropedic Surgery*—Drs. Wm. Watson, John Bell, Wm. Cornes.

*On Malignant Diseases*—Drs, S. H. Sawyers, J. C. Lay, J. H. Ealy.

*On Obstetrics*—Drs. H. L. Whitman, W. J. Robertson, A. C. Moon.

*On Meteorological and Medical Topography*—Drs. Philip Harvey, S. D. Richardson, A. T. Hudson, Wm. Watson, H. T. Cleaver, S. H. Sawyers, M. Cousins, J. Williamson, J. M. Shaffer, J. C. Shrader, A. G. Field.

Volunteer reports of important cases and interesting facts in either medicine or surgery will also be in order; and inasmuch as the Publishing Committee have thought best to defer the publication of the proceedings for 1868, the proceedings for 1868 and 1869 will probably be embodied in one volume, which will no doubt be alike creditable to the Society and valuable to the profession.

Beside these reports there is much yet to do in perfecting our Society organization, and establishing it upon a basis of the greatest possible usefulness and perpetuity. The next meeting, therefore, may be anticipated as one of unusual interest and importance. In behalf of those who shall be in attendance, an effort will be made for a reduction of the usual rates of fare on all the railroads of the State.

PHILIP HARVEY, M. D., *President*, Burlington.

A. G. FIELD, M. D., *Secretary*, Des Moines.

